# Meju, unsalted soybeans fermented with *Bacillus subtilis* and *Aspergilus oryzae*, potentiates insulinotropic actions and improves hepatic insulin sensitivity in diabetic rats

**DOI:** 10.1186/1743-7075-9-37

**Published:** 2012-05-02

**Authors:** Hye Jeong Yang, Dae Young Kwon, Min Jung Kim, Suna Kang, Sunmin Park

**Affiliations:** 1 Food Functional Research Division, Korean Food Research Institutes, 1201-62 Anyangpangyo-ro Bundang-Gu, Sungnam, 463-746, South Korea; 2 Dept of Food and Nutrition, Basic Science Institutes, Hoseo University, 165 Sechul-Ri, BaeBang-Yup, Asan-Si, ChungNam-Do, 336-795, South Korea

**Keywords:** Long-term fermented soybeans, Insulin sensitivity, Insulin secretion, β-cell proliferation, Hepatic glucose output

## Abstract

**Background:**

Although soybeans have the ability to attenuate insulin resistance, it is insufficient to alleviate type 2 diabetic symptoms and different types of fermented soybeans may have even better anti-diabetic effects. Meju, unsalted fermented soybeans exhibited better insulin sensitizing and insulinotropic actions than unfermented cooked soybeans (CSB). We investigated whether meju fermented in the traditional (TMS) manner for 60 days and meju fermented in the standardized (MMS) method inoculating *Bacillus subtilis* and *Aspergillus oryzae* for 6 days modulated insulin resistance, insulin secretion, and pancreatic β-cell growth and survival in 90% pancreatectomized (Px) diabetic rats, a moderate and non-obese type 2 diabetic animal model.

**Methods:**

Diabetic rats were divided into 3 groups: 1) TMS (n = 20), 2) MMS (n = 20) or 3) casein (control; n = 20). Rats were provided with a high fat diet (40 energy % fat) containing assigned 10% meju for 8 weeks. At the end of experiment insulin resistance and insulin secretion capacity were measured by euglycemic hyperinsulinemic clamp and by hyperglycemic clamp, respectively. Additionally, β-cell mass and islet morphohometry were determined by immunohistochemistry and insulin signaling in the liver was measured by western blot.

**Results:**

TMS and MMS increased isoflavonoid aglycones much more than CSB. CSB and TMS/MMS improved glucose tolerance in diabetic rats but the mechanism was different between treatments (P < 0.05). CSB enhanced peripheral insulin sensitivity including hepatic insulin sensitivity better than the control but TMS and MMS enhanced only hepatic insulin sensitivity through activating insulin signaling in diabetic rats (P < 0.05). However, TMS and MMS, but not CSB, potentiated glucose-stimulated insulin secretion and β-cell mass (P < 0.05). MMS had better insulinotropic actions than the control (P < 0.05).

**Conclusions:**

The anti-diabetic action of MMS, especially when fermented with *Bacillus subtilis* and *Aspergillus oryzae,* was superior to CSB by increasing isoflavonoid aglycones and small peptides with regard to type 2 diabetic rats.

## Introduction

Glucose homeostasis is maintained by the hyperbolic relationship of insulin sensitivity and insulin secretion. When insulin secretion can compensate for insulin resistance, normoglycemia can be maintained [1]. Type 2 diabetes develops if the compensation is impaired. Thus, the attenuation of insulin resistance and potentiation of insulin secretion are required to prevent type 2 diabetes. Anti-diabetic medications and functional foods need to improve insulin sensitivity and/or β-cell function and mass, and some herbs have been reported to have this capacity.

Soybeans (*Glycine max* MERILL) have been consumed as an important protein source to complement grain protein in Asian countries over a long period of time. Besides soy protein, they contain various nutritious and functional components such as isoflavonoids, which are helpful in protecting against metabolic diseases such as obesity and type 2 diabetes [2]. Some fermented soybeans such as chungkookjang and kochujang have been reported to have better anti-diabetic effects than unfermented soybeans in diabetic animals and humans [3,4]. Deonjang, soy sauce and kochujang are made with meju, long-term fermented soybeans, salts and other ingredients depending on the products. Since these fermented soybeans contain a lot of salt, they are difficult to develop into functional foods. Meju has not been consumed as it is due to being very dry and being no taste and bad odor. However, meju that is made through a process involving the long-term fermentation of unsalted soybeans with the *Bacillus* species and *Aspergillus* species may be a good candidate as a functional food for alleviating diabetes—if its anti-diabetic action can be established. Our previous cell-based study showed that methanol (M-60) and water (W-60) extracts from meju that was traditionally fermented for 60 days had a better insulin sensitizing action via activating PPAR-γ in 3T3-L1 adipocytes than unfermented soybeans [5]. M-60 and W-60 had greater glucose-stimulated insulin secretion capacity and greater β-cell viability than unfermented soybeans in insulinoma Min6 cells. These effects were associated with increased isoflavonoid aglycone such as genistein and daidzein and small peptides in the M-60 and W-60 of meju, respectively. The effects produced by traditionally made meju, where the fermentation process lasts for 60 days, were greater and more beneficial with regard to insulin-stimulated glucose uptake and glucose-stimulated insulin secretion than meju that was made after only 20 days of fermentation. Thus, fermentation periods and the kinds of microorganisms residing in soybeans may affect changes in isoflavonoids and peptides and so alter the anti-diabetic action.

We hypothesized that the 8-week consumption of meju, made both in the traditional and standardized manners, improved glucose homeostasis better than unfermented soybeans in diabetic rats and that the two different kinds of meju had similar effects. Over a long period of time we investigated the insulin sensitizing and insulinotropic actions of meju made in both the traditional and standardized manners on diabetic animals. In the present study, we used the 90% pancreatectomized (Px) rat model with high fat diet that is a well established model of type 2 diabetes which is especially applicable to Asian type 2 diabetes. Asians, especially from northeast Asia, have a low insulin secretory capacity and develop diabetes with little or no hyperglycemia prior to the failure of glycemic control [6]. However, both Western and Asian type 2 diabetes are characterized by insulin resistance combined with insufficient compensatory insulin secretion to maintain normal glucose control. The 90% pancreatectomized model is more similar to the etiology of the Asian form of type 2 diabetes with the surgery resulting in impaired insulin secretion, but with diet-induced insulin resistance developing simultaneously or shortly thereafter. The end result of surgical and dietary procedures is a rat type 2 diabetes with an etiology that resembles Asian type 2 diabetes, but that is consistent with both Western and Asian type 2 diabetes once they have developed. Since the Px rats release insulin sufficient not to induce ketosis, they are type 2 (not type 1) diabetic model. They are non-obese. To our best knowledge, this is the first report on the anti-diabetic activity of meju, long-term fermented soybeans with *Bacillus subtilis* and *Aspergillus oryzae* in a type 2 diabetic animal model.

## Materials and methods

### Preparation of meju

Meju was generated either by the traditional processing method or the standardized method. Soybeans were sorted, washed, and soaked in water for 12 h at 15°C and boiled for 4 h at 100°C. Cooked soybeans are formed into box-shaped blocks and fermented outdoors by micro-organisms naturally present in the environment for 60 days. The traditionally made meju is fermented primarily by the *Bacillus* species during the early stages of fermentation, followed by the *Aspergillus* species, which predominates during the remaining fermentation period, and the *Aspergillus oryzae* is the major microorganism in the final meju product. To make standardized meju, cooked soybeans were inoculated with *Bacillus subtilis* and formed into box-shaped blocks and the blocks were dried at 60°C for 24 h. They dried blocks were inoculated with *Aspergillus oryzae* and fermented in a fermentation chamber at 30°C for 6 days.

### Isoflavonoid and peptide contents of meju

The lyophilized unfermented cooked soybeans, meju made with traditional meju and meju made with standardized manner were extracted in 70% methanol containing 0.1% acetic acid and isoflavonoids in the supernatant of the extracts were detected using HPLC (PU 980, JASCO, Japan) equipped with an ODS A303 (4.6 × 250 mm, 5 μm, YMC, USA) reversed phase column and monitored at a wavelength of 254 nm with a UV detector. Elution was carried out at a flow rate of 1.0 mlmin^-1^ with water and acetonitrile containing 0.1% acetic acid. Peaks in each extract were identified by comparing them to 12 reference isoflavonoids purchased from Sigma Co. (St. Louise, MO) and Fujico (Tokyo, Japan).

The peptide contents of cooked soybeans or two kinds of meju made in the traditional manner or standardized manner were quantified using a ninhydrin method. Briefly, each water extract from the unfermented soybeans and meju was delipidated with chloroform and the water fractions were precipitated with 0.1 N NaOH at 110°C for 24 h. After adding 30% acetic acids into the precipitates for 6 min, the neutralized precipitates were reacted with a ninhydrin solution for 15 min and 50% ethanol was added and dissolved. The color changes of the solutions were measured at 570 nm at Spectrophotometer (Perkin-Elmer, Waltham, MA) and quantified with an external standard, L-leucine. The profiles of the peptides were determined by ultra performance liquid chromatography (UPLC, Waters Co.) using Acquity UPLC BEH C_18_ (2.1 × 100 mm, 1.7 μm; Waters, Milford, MA, USA) and monitored at a wavelength of 220 nm using a PDA detector. Elution was carried out at a flow rate of 0.35 mlmin^-1^ with a gradient solution of 0.1% trifluroacetic acid in water and 0.1% trifluroacetic acid in acetonitrile.

### Animals and diets

Male Sprague Dawley rats, weighing 211 ± 14 g, were housed individually in stainless steel cages in a controlled environment (23°C and a 12 hour light and dark cycle). All surgical and experimental procedures were performed according to the guidelines of the Animal Care and Use Review Committee at Hoseo University, Korea. The rats had a 90% pancreatectomy using the Hosokawa technique [7] or received a sham pancreatectomy (Sham). Px rats included in the experiments showed characteristics of type 2 diabetes, while the Sham rats did not. After removing 90% of the pancreas, it regenerates to about 50% of the original mass in approximately 2 weeks, after which there is no further regeneration. As a result, 90% pancreatectomy (Px) results in an about 50% decrease in insulin secretory capacity. This procedure combined with a high fat diet is well documented to result in insulin resistance with insufficient compensatory insulin secretion to maintain normal glucose control, which is consistent with type 2 diabetes. Thus, Px rats well represent type 2 diabetic animal model.

Px rats were randomly assigned to four different groups (control, CSB, TMS and MMS) of 20 animals according to dietary protein source. Px diabetic rats as a control group and sham-operated non-diabetic rats as a normal control were fed a casein diet. All rats freely consumed water and corresponding diets for 8 weeks. The diets were semi-purified, modifying a base AIN-93 formulation for the experimental diets. The CSB, TMS and MMS diets were also comprised of 10% lyophilized cooked soybeans, traditionally made meju or standardized meju, respectively. Since cooked soybeans and meju contained a mixture of carbohydrates, protein, and lipids, their compositions were analyzed. According to the results of the nutrient analysis of soybeans and meju, the macronutrient composition was tailored to exhibit equal proportions in all diets by adding soybean oil and cellulose. The protein sources of the CSB, TMS and MMS groups came from cooked soybeans, traditionally made meju and meju made in a modern manner, respectively, and any insufficiency in the quantity of protein in each diet was made up for with casein while the protein source of the control group was casein. All diets approximately consisted of 40 energy percent (En%) carbohydrates, 20 En% protein, and 40 En% fats (Table 1). The differences among these diets were essentially the degree of hydrolysis of protein and the presence of isoflavone, mainly as glycosides or aglycones.

**Table 1:** Composition of experimental diets

	Casein diet	Cooked soybean (CSB) diet	Meju (TMS/MMS) diet
Carbohydrates (Energy %)	39.6	40.0	39.9
Protein (Energy %)	19.9	19.6	19.4
Fat (Energy %)	40.5	40.5	40.8
Fiber (%)	8.9	8.9	8.9
Total isoflavonoids (%)	–	0.023	0.011/0.014
Isoflavonoid aglycones (%)	–	0.0004	0.005/0.01

### Oral glucose tolerance test (OGTT)

An oral glucose tolerance test was performed in the sixth-week in overnight-fasted animals by orally administering 2 g glucose/kg body weight. Blood samples were taken by tail bleeding at 0, 10, 20, 30, 40, 50, 60, 70, 80, 90, and 120 min after glucose loading, and serum glucose and insulin were measured with a Glucose Analyzer II (Beckman, Palo Alto, CA) and radioimmunoassay kit (Linco Research, Billerica, MA), respectively. The averages of the total areas under the curves for serum glucose and insulin were calculated by the trapezoidal rule. Since baseline values of serum glucose and insulin were not significantly different among the groups, their baseline values were not considered in the calculation of the areas. Overnight-fasted serum leptin and non-esterified fatty acid (NEFA) levels were measured by radioimmunoassay kit (Linco Research) and NEFA C enzymatic kit (Waco Diagnostics, Richmond, VA), respectively.

### Euglycemic hyperinsulinemic clamp

After catheterisation of the right carotid artery and left jugular vein in the 7th week, a euglycemic hyperinsulinemic clamp was performed on fasted conscious rats (n = 10) to determine insulin resistance as previously described [8]. [3-^3^H] glucose (Perkin Elmer, Wellesley, MA) was continuously infused during a four-hour period at the rate of 0.05 μCi/min. Basal hepatic glucose output was measured in blood collected at 100 and 120 minutes after initiation of the [3-^3^H] glucose infusion. A primed continuous infusion of human regular insulin (Humulin; Eli Lilly, Indianapolis, IN) was then initiated at a rate of 20 pmol kg^–1^ min^–1^ to raise plasma insulin concentration to approximately 1100 pM after 210–240 min. Blood samples from arteries were collected at 10-minute intervals for glucose evaluation, and 25% glucose was infused as needed to clamp glucose levels at approximately 6 mM. Disintegrations per min (dpm) of plasma [3-^3^H]-glucose with and without drying were measured; plasma concentration of ^3^H_2_O was determined by the difference between ^3^H counts with and without drying. Rates of whole body glucose uptake and basal glucose turnover were determined according to the ratio of the [3-^3^H] glucose infusion rate to the specific activity of plasma glucose (dpm/mmol) during the final 30 minutes. Hepatic glucose production at the hyperinsulinemic clamped state was determined by subtracting the glucose infusion rate from the whole body glucose uptake. After clamp, the rats were immediately anesthetised with a mixture of ketamine and xylazine and were killed by decapitation. Tissues were rapidly collected, weighed, frozen in liquid nitrogen, and stored at −70°C for further experiments.

### Hyperglycemic clamp

After seven weeks of treatment, catheters were surgically implanted into the right carotid artery and left jugular vein of ten conscious and overnight fasted rats from each group after anesthetization with ketamine and xylazine (100 mg and 10 mg/kg body weight, respectively). After 5–6 days of implantation, a hyperglycemic clamp was performed in free-moving and overnight fasted rats to determine insulin secretion capacity as described in previous studies [8]. During the clamp, glucose was infused to maintain serum glucose levels of 5.5 mM above the baseline and serum insulin levels were measured at 0, 2, 5, 10, 60, 90 and 120 min. After the clamp, rats were freely provided with foods and water for 2 days, and on the next day they were deprived of food for 16 hours. The rats were anesthetized with a mixture of ketamine and xylazine, and human regular insulin (5 U/kg body weight) was injected through the inferior vena cava of the rats. Ten min later, they were killed by decapitation and tissues were rapidly collected, frozen in liquid nitrogen, and stored at −70°C for further determinations. In order to determine the glycogen content in the liver, lysates were centrifuged at 3000 rpm for 10 minutes and the supernatants deproteinized with 1.5 N perchloric acid. The glycogen content was calculated from glucose from glycogen hydrolyzed by α-amyloglucosidase in an acid buffer [9]. Triglyceride was extracted with chloroform-methanol (2:1, vol/vol) from the liver and resuspended in pure chloroform [10]. After evaporating the chloroform, the residue was suspended with PBS with 0.1% triton X-100 and sonicated and boiled for 5 min. The triglyceride contents of the suspension were determined using a Trinder kit (Young Dong Pharm., Seoul, Korea).

### Immunoblot analysis

The livers taken from four rats after hyperglycemic clamp were used for an immunoblotting assay [8]. The frozen livers from each rat were lysed with a 20 mM Tris buffer (pH 7.4) containing 2 mM EGTA, 137 mM NaCl, 1% NP40, 10% glycerol, and 12 mM α-glycerol phosphate and protease inhibitors. After measuring protein contents in lysates (Biorad kit, Hercules, CA), equal amounts of protein in the lysates (30–50 μg) were resolved by SDS-PAGE and immunoblotted with phospho-Akt^ser478^, Akt, phospho-AMPK^thr172^, AMPK (Cell Signaling Technology, Beverly, MA), and phosphoenolpyruvate carboxykinase (PEPCK), generously provided by Dr. Granner of Vanderbilt University. The primary antibody was diluted with 1000X and secondary antibody was diluted with 5000X. The intensity of protein expression was determined using Imagequant TL (Amersham Biosciences, Piscataway, NJ).

### Immunohistochemistry and islet morphometry

At the end of the 8-week experimental period, nine to ten rats from each group were injected with BrdU (100 μg/kg body weight). Six hours post-injection, rats were anesthetized with intraperitoneal injections of mixture of ketamine and xylazine, and the pancreas was immediately dissected. The pancreas was fixed with 4% paraformaldehyde and paraffin-embedded, as described in previous studies [8,11]. Two serial 5-μm paraffin-embedded tissue sections were selected out of the seventh or eighth section to avoid counting the same islets twice when measuring the β-cell area, BrdU incorporation, and apoptosis. Endocrine β and α-cells were identified by applying guinea pig anti-insulin and rabbit anti-glucagon antibodies to the sections. BrdU incorporation in β-cells was determined by staining rehydrated paraffin sections with anti-insulin and anti-BrdU antibodies [11]. Apoptosis of β-cells was measured by TUNEL kit (Roche Molecular Biochemicals, Indianapolis, IN) and counterstained with hematoxylin and eosin to visualize islets [8].

The pancreatic β-cell area was measured by examining all of the non-overlapping images in two insulin-stained sections of each rat at a magnification of 10x with a Zeiss Axiovert microscope (Carl Zeiss Microimaging, Thornwood, New York). The results of β-cell quantification were expressed as the percentage of the total surveyed area containing insulin-positive cells, measured by IP Lab Spectrum software (Scanalytics Inc., Fairfax, VA). Pancreatic β-cell mass was calculated by multiplying the percentage of insulin-positive area by the weight of the corresponding pancreatic portion [8,12]. The individual β-cell size was determined as the insulin-positive area divided by the number of nuclei counted in the corresponding insulin-positive structures in randomly immunofluoresence-stained sections. Enlarged individual β-cell size indicates the induction of β-cell hypertrophy [8]. Beta-cell proliferation was calculated as the total BrdU^+^ nuclei in β-cell nuclei per pancreas section while apoptosis of β-cells was measured by the total number of apoptotic bodies in β-cell nuclei per pancreas section [8,12].

#### *Statistical analysis*

Statistical analysis was performed using SAS software and all results expressed as mean ± standard deviation. The biological and metabolic effects of casein (control), CSB, TMS, and MMS were compared by one-way ANOVA. Pearson’s correlation coefficients were determined between isoflavonoid aglycone contents the rats in the diet and some parameters. Significant differences in the main effects among the groups were identified by Tukey’s test at *P* < 0.05. The differences between the Px diabetic rats (control) and Sham non-diabetic rats (normal control) were determined by two-sample t test.

## Results

### Isoflavonoids and peptide contents

Isoflavonoid glycosides decreased in MMS and TMS in comparison to CSB, unfermented soybeans, while isoflavonoid aglycones increased in an ascending order of CSB, TMS and MMS (Table 2). Daidzein, glycitein, and genistein increased in MMS the most when fermentation was carried out with *Bacillus subtilis* and *Asperilus oryzae*. Peptide contents were reduced by fermentation and the reduction was greater in TMS than MMS (Table 2).

**Table 2:** Isoflavonoid contents and peptides (μg/g of dry matter)

	CSB	TMS	MMS
Total isoflavonoid glycosides	1,843 ± 23^a^	644 ± 134^b^	432 ± 76^b^
Total isoflavonoid aglycosides	23±3^c^	274 ± 48^b^	673 ± 103^a^
Daidzein	trace	112 ± 17^b^	198 ± 65^a^
Glycitein	7 ± 2^c^	25 ± 3^b^	83 ± 27^a^
Genistein	16 ± 2^c^	137 ± 22^b^	392 ± 76^a^
Peptides (mg/ g)	48.0 ± 5.9^a^	3.2 ± 1.1^c^	21 ± 3.5^b^

CSB, cooked soybeans; TMS, traditionally made meju; MMS, meju made with a standard method. ^a,b,c^ Means of the same row without a common letter significantly differ at P < 0.05 by Tukey test.

### Body weight and overnight-fasting glucose, insulin, leptin and NEFA

Px diabetic rats had a lower body weight than Sham rats although Px rats consumed more calories daily than Sham rats, possibly due to urinary glucose excretion and insulin insufficiency since our preliminary study showed that urinary glucose was detected after meal and serum insulin levels were lowered in Px rats than Sham rats. Px rats However, epididymal fat pads were not significantly different between Px and Sham rats and serum leptin levels were also not significantly different. Among Px rats, CSB, MMS and TMS displayed comparable body weight gain but MMS and TMS had less epididymal fat pads than the control and lower caloric intake (Table 3). However, serum leptin levels were not significantly different among groups.

**Table 3:** Physiological characteristics

	Control (n = 20)	CSB (n = 20)	MMS (n = 20)	TMS (n = 20)	Sham rats (n = 20)
Body weight (g)	310.9 ± 30.3	299.8 ± 54.8	320.9 ± 43.3	321.6 ± 23.5	382.4 ± 26.7^†^
Epididymal fat pads (g)	3.4 ± 0.7^a^	3.2 ± 0.7^a^	2.5 ± 0.6^b^	2.7 ± 0.6^b^	3.2 ± 0.7
Food intake (g/day)	17.6 ± 2.1^a^	17.1 ± 1.9^a^	14.1 ± 1.7^b^	15.1 ± 1.8^b^	14.6 ± 2.8^†^
Serum leptin (ng/mL)	3.2 ± 0.6	3.0 ± 0.5	3.1 ± 0.6	3.2 ± 0.7	5.3 ± 0.9
Serum glucose (mM)	8.3 ± 0.9^a^	7.5 ± 0.8^b^	6.5 ± 0.9^c^	6.6 ± 0.9^c^	4.7 ± 0.7^†^
Serum insulin (ng/mL)	0.53 ± 0.07^b^	0.55 ± 0.08^b^	0.68 ± 0.09^a^	0.65 ± 0.09^a^	0.72 ± 0.14^†^
Serum non- esterified fatty acids (μM)	921 ± 117^a^	802 ± 104^b^	702 ± 92^c^	705 ± 93^c^	635 ± 83^††^

Values are mean ± SD. The control group was fed a casein diet, CSB group fed a cooked soybean diet, MMK group fed a diet containing 10% meju made with standard method, and TMK group fed a diet containing 10% meju made with traditional method.

^a,b^ Means of the same row without a common letter significantly differ at P < 0.05 by Tukey test.

^†^Significantly different from Px control at P < 0.05.

Hyperglycemia in Px rats was due to a concomitant decrease in serum insulin levels (Table 3). As serum glucose levels represent a combination of insulin resistance and insulin secretion, CSB had decreased serum glucose levels without serum insulin levels changing in comparison to the control. This decrease in the CSB group was not as much as that in the MMS and TMS groups. However, MMS and TMS had lowered serum glucose levels as a result of increasing serum insulin levels in comparison to the control (Table 3). Overnight-fasted serum NEFA levels increased among Px rats more than Sham rats. Similar to serum glucose levels, serum NEFA levels were lowered in the descending order of control, CSB, TMS, and MMS groups among Px rats (Table 3).

### Area under the curve of glucose and insulin in OGTT

**Figure 1:**
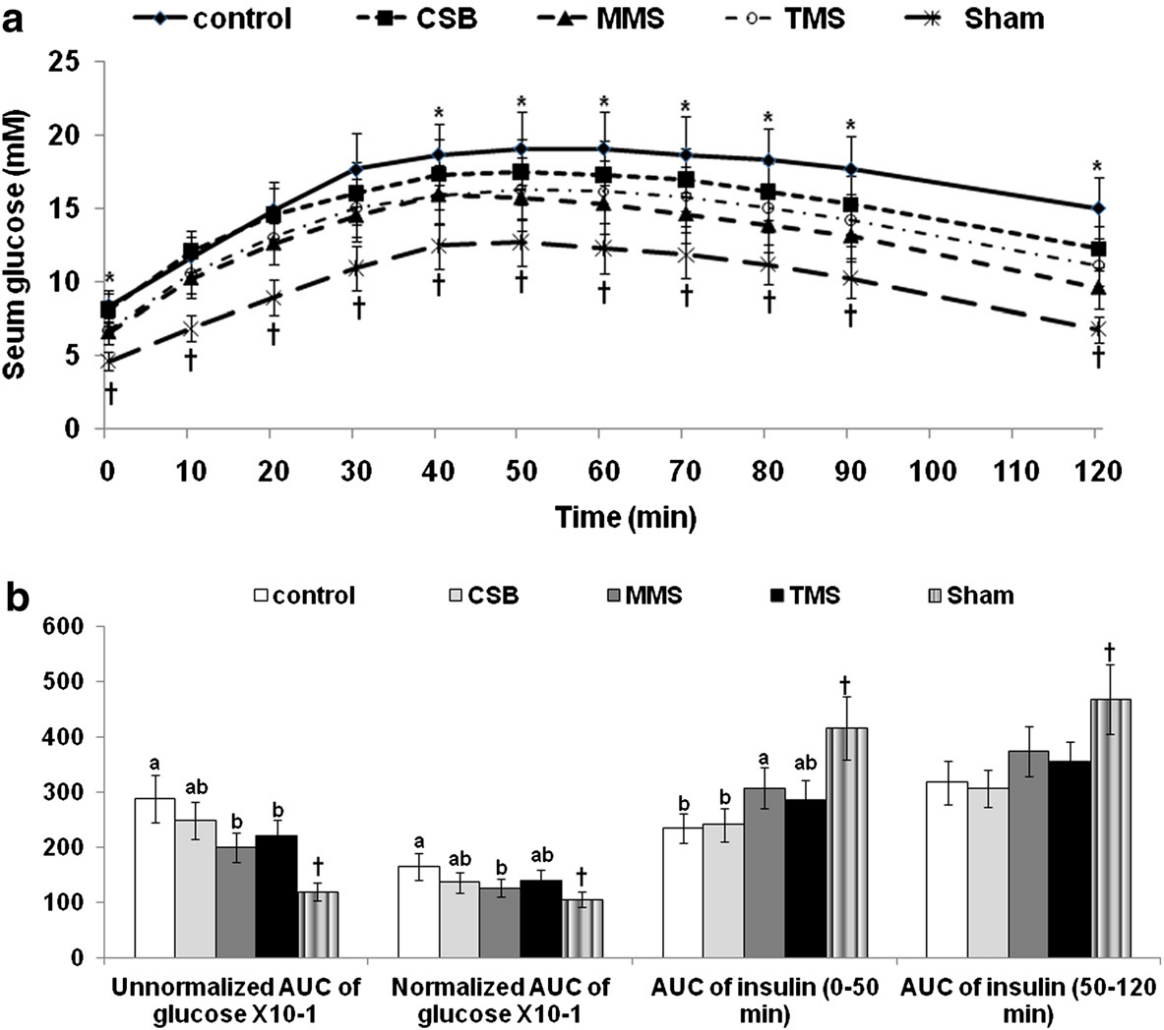
**The changes in serum glucose levels and the area under the curve for serum glucose and insulin during oral glucose tolerance testing**. Oral glucose tolerance tests were performed on Px rats fed diets containing 10% cooked soybeans (CSB), meju made in the traditional (TMS) or standardized (MMS) manner, or casein (control), for 8 weeks following oral loading with 2 g glucose per kg body weight. Blood samples were taken at the time points indicated, serum glucose levels (A) were measured, and the area under the curve for glucose and insulin was calculated (B). The sample size in each group was the same as in Table 3. ^*^Significantly different among groups of Px rats at P < 0.05. ^a,b^ Means of the bars without a common letter significantly differ at P < 0.05 by Tukey test. ^†^Significantly different from Px control at P < 0.05.

After the oral glucose load, Px rats increased serum glucose levels much greater than Sham rats: the peak of serum glucose levels was higher in Px rats than Sham rats and serum glucose levels decreased slowly from the peak in Px rats in comparison to Sham rats (Figure 1A). Px rats of the MMS and TMS groups exhibited lower serum glucose levels at the peak than those of the control and the serum glucose levels decreased slowly among the rats in the MMS and TMS groups. As a result, TMS and MMS diets among Px rats produced significantly lower areas under the curve for serum glucose levels by 30 and 24 %, respectively, compared with the casein diet (Figure 1B). After normalizing serum glucose levels at each time point with fasting serum glucose levels during OGTT, the calculated area under the curve decreased by 22.9 and 14.4 %, respectively. This decrease in MMS and TMS was not, however, as low as that in Sham rats. Thus, Px rats in the MMS and TMS groups had improved glucose tolerance relative to Px rats in the control group (Figure 1B).

After challenging glucose load, serum insulin levels increased the first part of OGTT and it was raised again in the second part. We separated the area under the curve of insulin at the 50 min since serum glucose levels reached the peak at 50 min in diabetic rats. In non-diabetic rats, serum glucose levels were peak at 20 min and serum insulin were peak at around 15–20 min after glucose challenge but the peak of serum glucose levels were delayed at 40–50 min in insulin resistant rats as well as diabetic rats [13]. Thus, area under the curve of insulin was calculated into two parts by the peak of serum glucose levels. Px diabetic rats displayed a lower area under the curve for serum insulin levels at both first and second phases than Sham non-diabetic rats (Figure 1B). MMS and TMS increased the area under the curve for insulin at the first phase in comparison to the control, but not the second phase (Figure 1B). Thus, the decrease in serum glucose levels in rats fed MMS and TMS was associated with increased serum insulin levels during OGTT, especially during the first phase. MMS displayed an improvement of glucose tolerance in comparison to TMS as a result of increased serum insulin levels.

### Insulin sensitivity measured by euglycemic hyperinsulinemic clamp

**Figure 2:**
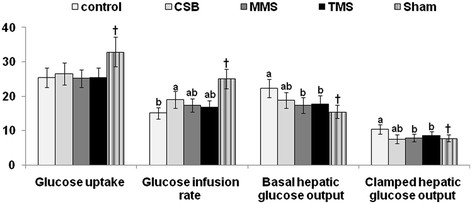
**Metabolic parameters during euglycemic hyperinsulinemic clamp.** After 8 weeks of treatment with four different diets—one containing 10% cooked soybeans (CSB), one containing meju made in the traditional (TMS), one containing meju made in the standardized (MMS) manner, and one containing casein (control)—euglycemic hyperinsulinemic clamp was performed in conscious, free moving and overnight fasted diabetic rats to determine whole body insulin resistance. Whole body glucose infusion rates (GIR) and glucose uptake and hepatic glucose output at basal and clamped states were investigated. The sample size in each group was the same as in Table 4. Means of the bars without a common letter significantly differ at P < 0.05 by Tukey test. ^†^Significantly different from Px control at P < 0.05.

As depicted in Figure 2, whole body glucose infusion rates and glucose uptake, representing peripheral insulin sensitivity, decreased in Px rats in comparison to Sham rats. In comparison with casein-fed rats, CSB significantly increased glucose infusion rates among Px rats but meju did not significantly elevate rates. However, MMS-fed rats seemed to have increased rates. In addition, insulin-stimulated glucose uptake, representing peripheral insulin resistance, was not significantly different among the groups of Px rats (Figure 2). Hepatic glucose output in basal and hyperinsulinemic clamped states was not suppressed among Px rats as much as it was among Sham rats (Figure 2). A good portion of the difference in glucose infusion rates among the groups could be accounted for by the insulin-stimulated reduction in hepatic glucose production, which represents attenuated hepatic insulin sensitivity. Basal hepatic glucose production was significantly lowered among the MMS and TMS groups than among the control group and hepatic glucose output in hyperinsulinemic states was also more suppressed among CSB, TMS and MMS groups than the control (Figure 2).

### Hepatic insulin signaling

**Figure 3:**
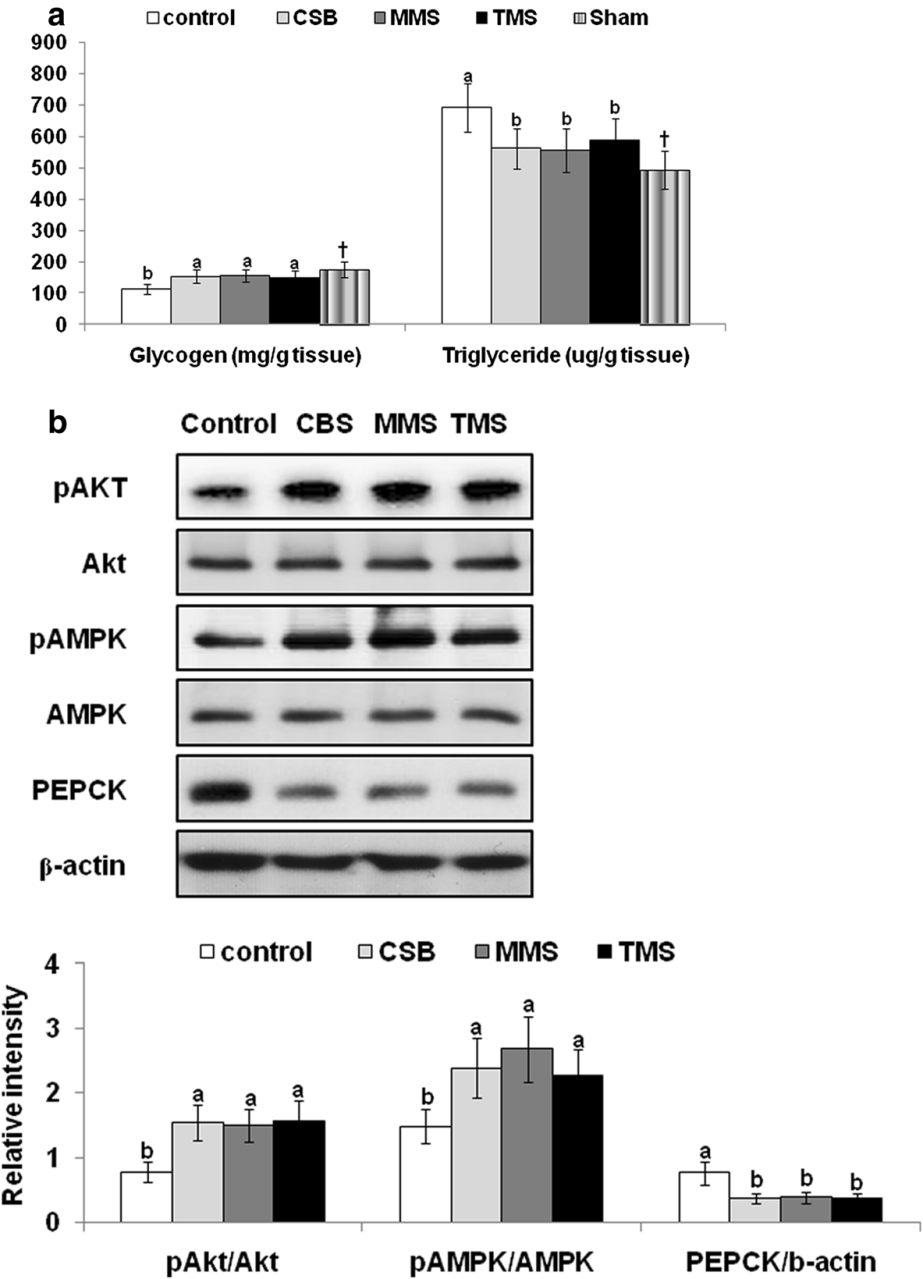
**Hepatic insulin signaling at the end of experiment**. The liver of Px rats injected with 5 U insulin/kg body weight into the inferior vena cava was collected after they were fed with diets containing 10% cooked soybeans (CSB), meju made with traditional (TMS) or standardized (MMS) manner, or casein (control) for 8 weeks. The glycogen and triglyceride contents in the liver were measured (A). In addition, the phosphorylation of Akt and AMPK and the expression of phosphoenolpyruvate carboxykinase (PECK) were measured by immunoblotting method (B). The sample size in each group was 4. ^a,b^ Means of the bars without a common letter significantly differ at P < 0.05 by Tukey test.

Liver glycogen also increased in the CSB, TMS and MMS groups but triglyceride accumulation exhibited a reverse or opposite trend to glycogen accumulation (Figure 3A). Serine phosphorylation of Akt was attenuated in Px rats fed a casein diet by 32% in comparison to Sham rats, while PEPCK expression was elevated among Px rats by 243% (Data not shown). CSB, MMS and TMS increased the phosphorylation of Akt compared to the control (Figure 3B). The protein levels of Akt showed no difference among the treatments. In parallel with the intensity of Akt phosphorylation, PEPCK expression was decreased (Figure 3B). Decreased expression of PEPCK indirectly implied that an enhanced insulin signaling cascade in the liver resulted in the suppression of hepatic glucose output during the hyperinsulinemic clamp state. In addition, the phosphorylation of AMPK was elevated in the Px rats fed CSB, TMS, and MMS in comparison to the control group (Figure 3B).

### First and second phase insulin secretion during hyperglycemic clamp

**Figure 4:**
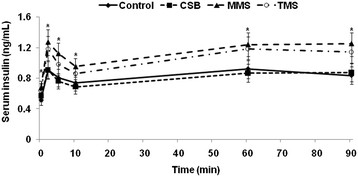
**Insulin secretion capacity during hyperglycemic clamp**. At the end of the experimental periods, hyperglycemic clamp was performed on Px rats fed diets containing 10% cooked soybeans (CSB), meju made in the traditional (TMS) or standardized (MMS) manner, or casein (control). During hyperglycemic clamp, serum insulin levels were measured in free-moving and overnight-fasted diabetic rats as serum glucose levels at 5.5 mM above fasting levels were maintained. The sample size in each group was the same as in Table 4. ^*^Significantly different among groups of Px rats at P < 0.05.

As insulin secretion is known to be biphasic during glucose load, serum insulin levels peaked at 2 to 5 min (first phase) and then declined to a nadir at 10 min and then increased and remained steady at 60–90 min (second phase) during hyperglycemic clamp. The serum insulin levels of Px rats were half those of Sham rats during first and second phase insulin secretion under hyperglycemic clamp (data not shown). MMS and TMS increased serum insulin levels during both first and second phase more than the control during hyperglycemic clamp (Figure 4). First and second phase insulin secretion was positively correlated with dietary isoflavonoid aglycone contents (r = 0.47 and r = 0.36, respectively). However, the increase in insulin secretion among MMS and TMS did not reach the level exhibited by Sham rats, which is shown in the area under the curve for insulin in Table 4. CSB did not alter insulin secretion in comparison to the control. The area under the curve for serum insulin levels during hyperglycemic clamp well reflected first and second phase insulin secretion (Table 4).

**Table 4:** Insulin secretion capacity during hyperglycemic clamp

	Control (n = 10)	CSB (n = 10)	MMS (n = 10)	TMS (n = 10)	Sham rats (n = 10)
Serum insulin at basal state (ng/mL)	0.51 ± 0.09^b^	0.57 ± 0.09^ab^	0.67 ± 0.10^a^	0.64 ± 0.11^a^	0.79 ± 0.11^†^
AUC of serum insulin at first phase	5.3 ± 0.8^c^	4.3 ± 0.7^d^	7.3 ± 1.1^a^	6.5 ± 1.2^b^	13.5 ± 1.8^†^
AUC of serum insulin at second phase	47.2 ± 6.7^b^	42.1 ± 6.4^b^	65.1 ± 9.4^a^	60.4 ± 8.6^a^	114.5 ± 16.5^†^
Glucose infusion rate (mg/kg bw/min)	7.5 ± 1.0^b^	7.4 ± 1·1^b^	10.1 ± 1.3^a^	9.4 ± 1.2^a^	16.1 ± 2.2^†^
Insulin sensitivity (μmol glucose · min-1 · 100 g-1 per μmol insulin/L)	25.5 ± 3.4^b^	29·3 ± 3.9^a^	24.2 ± 2.9^b^	24.2 ± 3·4^b^	16.8 ± 2.3^†^

Values are mean ± SD. The control group was fed a casein diet, CSB group fed a cooked soybean diet, MMK group fed a diet containing 10% meju made with standard method, and TMK group fed a diet containing 10% meju made with traditional method. AUC of first phase insulin secretion was defined as the area under the curve (AUC) of serum insulin levels from 2 to 10 mins, with second phase from 60 to 120 mins. Insulin sensitivity at hyperglycemic state was calculated as the ratio of glucose infusion rate to steady-state plasma insulin levels.

^a,b^ Means of the same row without a common letter significantly differ at P < 0.05 by Tukey test.

^†^Significantly different from Px control at P < 0.05.

Glucose infusion rates during hyperglycemic clamp indicated β-cell function and insulin sensitivity at hyperglycemic state, calculated as the ratio of glucose infusion rate to steady-state serum insulin levels [14]. Glucose infusion rates were higher in Sham rats than Px rats but insulin sensitivity in a hyperglycemic state was not significantly different between Sham rats and Px rats. The MMS and TMS groups of Px rats had increased glucose infusion rates during hyperglycemic clamp in comparison to the control group but the rates did not reach the levels exhibited by the Sham rats (Table 4). In addition, insulin sensitivity in a hyperglycemic state was not significantly different among the groups of Px rats, although it appeared to be slightly higher in the CSB group (Table 4). These results indicated that MMS and TMS supplementation rectified the impairment of glucose intolerance by improving β-cell function rather than increasing insulin sensitivity in Px rats.

### Pancreatic β-cell mass, proliferation and apoptosis

The percentage of the pancreatic β-cell area was greater in Px rats than Sham rats as islets were replenished in greater numbers than had existed in the original state when the pancreas was regenerated by up to 50–60% in comparison to the original pancreas as a result of increased proliferation and neogenesis after 90 % of the pancreas had been removed. However, pancreatic β-cell mass, calculated by multiplying β-cell area by the pancreas weight, was greater in Sham rats than Px rats since the pancreas size of Px rats was 50–60% of the Sham rats. Pancreatic weight was significantly higher in Sham rats than Px rats by about 2 folds while it was not significantly different among the groups of Px rats (data not shown). Only MMS increased the percentage of β-cell area more than the control. Although pancreas weight was not significantly different among the differently treated Px rats, total β-cell mass was higher among MMS treated Px rats in comparison to the other groups of Px rats (Table 5). Total β-cell mass was positively correlated with isoflavonoid aglycone contents in diets (r = 0.38). Individual β-cell size was greater in Px rats than Sham rats, indicating that β-cell displayed hypertrophy in Px rats. MMS and TMS increased β-cell area by increasing the number of β-cells (hyperplasia) by enhancing proliferation and reducing apoptosis in Px rats (Table 5). However, MMS and TMS reduced the individual β-cell size in Px rats, suggesting recuperation from hypertrophy (Table 5). In addition, the ratio of β-cells and α-cells was higher in the MMS and TMS groups compared to the control group (Table 5). Thus, MMS and TMS produced better insulin secretion patterns during hyperglycemia, which may be associated with elevated β-cell mass though increased hyperplasia.

**Table 5:** The modulation of islet morphometry

	Control (n = 7)	CSB (n = 7)	MMS (n = 7)	TMS (n = 7)	Sham rats (n = 7)
β-cell area (%)	6.6 ± 0.8^b^	7.2 ± 0.9^ab^	7.8 ± 0.9^a^	7.4 ± 0.9^ab^	5.7 ± 0.7^†^
Individual β-cell size (μm^2^)	226.2 ± 32.8^a^	207.7 ± 29.7^ab^	176.3 ± 28.1^b^	210.4 ± 30.6^ab^	174.6 ± 29.8^†^
Absolute β-cell mass (mg)	20.6 ± 3.2^b^	22.7 ± 3.6^ab^	25.2 ± 3.9^a^	23.5 ± 3.7^ab^	30.9 ± 4.2^††^
BrdU^+^ cells (% BrdU^+^ cells of islets)	0.86 ± 0.11^b^	0.94 ± 0.13^ab^	1.11 ± 0.14^a^	0.99 ± 0.13^a^	0.69 ± 0.10^†^
Apoptosis (% apoptotic bodies of islets)	0.69 ± 0.09^a^	0.64 ± 0.08^ab^	0.59 ± 0.08^b^	0.62 ± 0.08^ab^	0.60 ± 0.08^†^
Ratio of β:α cells	4.7 ± 0.6^b^	5.4 ± 0.7^ab^	5.9 ± 0.8^a^	5.6 ± 0.8^a^	5.2 ± 0.9

Values are mean ± SD. The control group was fed a casein diet, CSB group fed a cooked soybean diet, MMK group fed a diet containing 10% meju made with standard method, and TMK group fed a diet containing 10% meju made with traditional method.

^a,b^ Means of the same row without a common letter significantly differ at P < 0.05 by Tukey test.

^†^Significantly different from Px control at P < 0.05. ^††^ at P < 0.01.

## Discussion

Koreans exhibited a lower prevalence of diabetes but the prevalence has been remarkably increased in recent years [15]. This may be due to several factors such as a low fat diet and a high consumption of soybeans. Soybean products, especially fermented soybean products, are used for meal preparation on a daily basis in Korea and their routine consumption may be helpful in preventing the development of diabetes [16,17]. Meju is a basic component of fermented soybean products such as deonjang, soy sauce and kochujang in Korea and it is made both in a traditional manner and a standardized manner: traditionally made meju is made by fermenting soybeans with local microorganisms for 60 days; and standardized meju is made by fermenting soybeans inoculating with *Bacilus subtilis* and *Aspergillus oryze* for 6 days. Our previous study revealed that meju traditionally fermented for 60 days has better anti-diabetic effects by enhancing insulin-stimulated glucose uptake and glucose-stimulated insulin secretion than meju fermented for shorter periods (20 days) in cell-based studies [5]. In addition, our preliminary study found that the contents of isoflavonoids in meju fermented for 60 days in the traditional manner were similar to those found in meju fermented for 6 days in the standard manner. In the present study, we found that TMS and MMS had similar antidiabetic effects in diabetic rats. Although MMS induced a more enhanced response than TMS, the difference was not significant. TMS and MMS potentiated β-cell function and mass more than the control among diabetic rats; and although they did not improve peripheral insulin resistance, they did enhance hepatic insulin sensitivity in comparison to the control.

It has been well documented in previous studies that the fermentation of soybeans increases isoflavone aglycones and that the aglycone forms have better bioavailability and functionality due to their enhanced absorption in humans and animals [18,19]. Although the latter contention is still somewhat controversial, the consumption of tempeh, a fermented soybean product containing mostly isoflavone aglycones, resulted in higher serum levels of daidzein and genistein than that induced by unfermented soybeans [20]. Several studies have reported that meju, long-term fermented soybeans, elevates the quantity of isoflavonoid aglycones to a much greater degree than chungkookjang, short-term fermented soybeans containing the *Bacillus* species [5,21]. The present study showed that isoflavonoid aglycones such as daidzein, glycitein and genistein occurred in large numbers in meju, especially MMS. In comparison to TMS, MMS elevated genistein more than daidzein and glycitein. This was consistent with Jang et al. [22]. These changes in isoflavonoid aglycones and peptide profiles according to fermentation processes may be related to preventive type 2 diabetes properties.

The potentiation of β-cell function and mass as a result of increasing proliferation and decreasing apoptosis may be related to increased isoflavonoid aglycones such as daidzein, genistein and glycitein in TMS and MMS in comparison to CSB. Previous studies have revealed that up to 20 μM genisitein improved glucose-stimulated insulin secretion in islets with β-cell proliferation and in insulinoma cells by augmenting cyclic adenosine 3′5′-monophosphate (cAMP) accumulation to activate PKA signaling in a dose dependent manner [23-25]. However, high dosages of genistein (100 μM) were reported to rather suppress insulin secretion by acting as a tyrosine kinase inhibitor [26]. However, the consumption of genistein did not reach 100 μM serum genistein levels and in most cases serum genistein levels do not exceed 5 μM in animals and humans [18-20]. Our previous study has also shown that relatively short-term fermented (43 h) soybeans, such as chungkookjang, potentiate glucose-stimulated insulin secretion and β-cell mass in diabetic rats [26]. In the previous study, higher contents (20%) of chungkookjang were included in the diet and the insulinotropic action was similar to meju (10%). This increase in β-cell mass can enhance glucose-stimulated insulin secretion since 90% pancreatectomozed rats exhibit insulin deficiency due to insufficient β-cell mass and the increased β-cell mass is associated with the potentiation of the insulin/IGF-1 signaling cascade in the islets of diabetic rats who have consumed chungkookjang [27]. Chungkookjang increases phosphorylation of cAMP responding element binding protein by elevating intracellular cAMP levels, so inducing the expression of IRS2, which is known as a key modulator of β-cell growth and survival [28]. Thus, TMS and MMS improved β-cell mass, possibly by enhancing insulin/IGF-1 signaling in a similar manner to chungkookjang. The potentiation of β-cell function and mass through enhancing insulin signaling was associated with increased isoflavonoid aglycones, especially genistein.

Previous studies have reported that soy protein produces lower fasting plasma glucose and insulin concentrations than a casein diet in non-diabetic animal and human studies but recent studies have shown that soybeans do not have a beneficial effect on glycemic control in diabetic humans [29-31]. In the present study, CSB—unfermented soybeans—improved insulin sensitivity but not insulin secretion capacity in diabetic animals. However, meju—long-term fermented soybeans—improved glycemic control mainly by potentiating insulinotropic actions but did not improve peripheral insulin resistance. TMS and MMS did not modulate whole body glucose uptake into peripheral tissues such as adipose tissues and skeletal muscles but MMS and TMS did improve hepatic insulin resistance by suppressing hepatic glucose output at basal and hyperinsulinemic clamp states. However, these results contradicted our cell-based study [5] that stated that methanol and water extracts increased insulin-stimulated glucose uptakes by activating PPAR-γ in 3T3-L1 adipocytes. In addition, our previous study revealed that chungkookjang, short-term fermented soybeans, enhances insulin sensitivity by increasing glucose uptake in skeletal muscles and glucose infusion rates better in diabetic rats [32]. This difference between chungkookjang and meju may involve peptide contents and profiles since the content of isoflavonoid aglycones was rather higher in meju, especially MMS, than in CSB, but peptide content was lower in TMS and MMS in comparison to CSB. Thus, changes involving peptides in meju may not affect the modulation of insulin resistance in diabetic rats. It is valuable to study the peptide profiles of chungkookjang and meju in order to determine what differences they cause in the response of diabetic animals to insulin resistance. However, like chungkookjang, meju decreases hepatic glucose output at hyperinsulinemic clamped states more than unfermented soybeans. This reduction in the liver size of rats that consumed meju or chungkookjang was associated with decreased phosphoenolpyruvate carboxykinase expression that resulted from improved hepatic insulin signaling via potentiating the serine^473^ phosphporylation of Akt. Thus, meju, long-term fermented soybeans, demonstrated that it could induce an improvement in hepatic insulin resistance in diabetic rats.

## Conclusion

The contents of isoflavonoid aglycones increased in an ascending order of CSB, TMS, and MMS. The isoflavonoid aglycones contents of MMS were much higher than CSB and TMS—by 29.3 and 2.5 folds, respectively. CBS, TMS and MMS improved glucose tolerance in diabetic rats while CSB did not enhance it as much as MMS and TMS. However, the mechanism of the improvement was different in CSB and MMS/TMS. CSB enhanced peripheral insulin sensitivity including hepatic insulin sensitivity better than the control but TMS and MMS enhanced only hepatic insulin sensitivity in diabetic rats. However, CSB did not improve glucose-stimulated insulin secretion and β-cell mass in diabetic rats while TMS and MMS did potentiate insulin secretion and β-cell mass. In addition, MMS had better insulinotropic actions than TMS. The results of our previous cell-based studies were not exactly consistent with those of the present animal study. The results suggested that MMS and TMS improved glycemic control by potentiating insulinotropic actions and alleviating hepatic insulin resistance in diabetic rats. MMS may be a good candidate as a functional food for relieving diabetes and also postmenopausal symptoms since it contains greater quantities of isoflavonoid aglycones, especially daidzein and genistein.

## Competing interests

The authors declare that they no competing interests.

## Authors’ contributions

HJY helped prepare meju and performed all statistical analysis of results; DYK performed to prepare different kinds of meju and to analyze their composition and helped draft the manuscript; MJK helped analyze the contents of isoflavonoids and peptides; ESH helped perform animal study; DSK performed all animal surgery and animal study; SMP overlooked all aspects of the projects, wrote the draft manuscript abd obtained the funding for the work. All authors read and approved the final manuscript.
